# Role of Dentistry in Global Health: Challenges and Research Priorities

**DOI:** 10.1177/0022034521992011

**Published:** 2021-02-04

**Authors:** F.N. Hugo, N.J. Kassebaum, W. Marcenes, E. Bernabé

**Affiliations:** 1 Departamento de Odontologia Preventiva e Social, Faculdade de Odontologia, Universidade Federal do Rio Grande do Sul, Porto Alegre, Brazil; 2 Institute of Health Metrics and Evaluation, University of Washington, Seattle, WA, USA; 3 Affordable Health initiative, London, UK; 4 Dental Public Health Group, Faculty of Dentistry, Oral and Craniofacial Sciences, King’s College London, London, UK

**Keywords:** global burden of disease, social determinants of health, healthcare disparities, universal health care, public health dentistry, dental care

## Abstract

Despite some improvements in the oral health of populations globally, major problems remain all over the planet, most notably among underprivileged communities of low- and middle-income countries but also in high-income countries. Furthermore, essential oral health care has been a privilege, instead of a right, for most individuals. The release of the *Lancet* issue on oral health in July 2019 built up some momentum and put oral conditions and dental services in the limelight. Yet, much work is still needed to bridge the gap between dental research and global health and get oral health recognized as a population health priority worldwide. Using the framework proposed by Shiffman, we argue that a global health network for oral health must be harnessed to influence global health policy and drive health system reform. We have identified challenges around 4 key areas (problem definition, positioning, coalition building, and governance) from our experience working in the global health arena and with collaborators in multidisciplinary teams. These challenges are outlined here to validate them externally but also to call the attention of interested players inside and outside dentistry. How well our profession addresses these challenges will shape our performance during the Sustainable Development Goals era and beyond. This analysis is followed by a discussion of fundamental gaps in knowledge, particularly in 3 areas of oral health action: 1) epidemiology and health information systems; 2) collection, harmonization, and rigorous assessment of evidence for prevention, equity, and treatment; and 3) optimal strategies for delivering essential quality care to all who need it without financial hardship.

## Introduction

Health systems around the world are now challenged by the increasing burden of noncommunicable diseases, the growing aging population (GBD 2017 SDG Collaborators 2018), and the rising health care costs associated with the adoption of technological innovations (Dieleman et al. 2017). The perspective of addressing common risk factors is reinforced in the Sustainable Development Goal (SDG) agenda (UN General Assembly 2015). The Global Burden of Disease (GBD) findings have shown that untreated common oral conditions remain a significant global health challenge, with evidence of inequalities in the burden of untreated caries, severe periodontitis, and total tooth loss by economic development (Bernabé et al. 2020). The GBD study was instrumental in providing data for advocacy with policy makers that oral conditions pose an important population challenge with significant economic impacts and must be included in the global health agenda (Righolt et al. 2018; Bernabé et al. 2020).

Against this backdrop, what is the role of the dental profession and dental research in global health? Global health is about reaching beyond borders, disciplines, and cultures to tackle health inequalities but also about preparing the health workforce and health systems to the effects of these threats of global dimension (Macfarlane et al. 2008; Holst 2020). Within this context, the dental profession has a role in addressing the high prevalence of oral conditions and the inequalities in the distribution of and access to dental care, as well as in preparing the workforce and systems for the impact of future threats that aligns with the goals of global health.

## Challenges in Global Oral Health

Much work is needed to bridge the gap between dental research and global health and to get oral health recognized as a population health priority worldwide. We assert that a global health network for oral health must be harnessed to influence global health policy and drive health system reform. Global health networks do matter, especially to shape the way that challenges and solutions are understood and to advocate for governments and international agencies to address them. As argued by Shiffman (2017, 2018), all global health networks face 4 key strategic challenges in generating attention and resources for the conditions that concern them (Figure).

**Figure.**
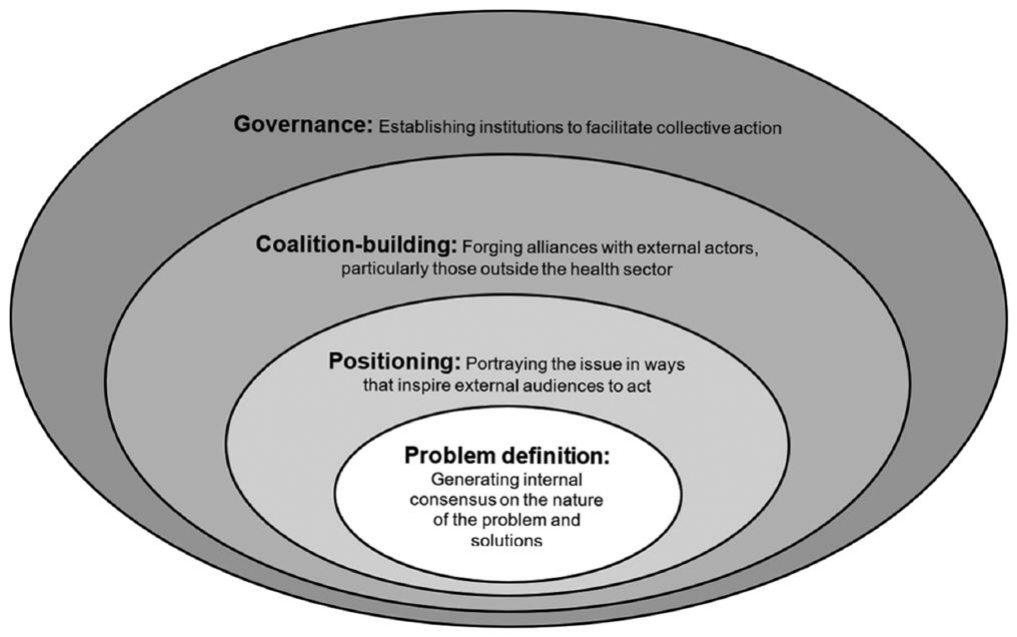
The 4 challenges faced by global health networks. Adapted from Shiffman (2017).

The first challenge is to reach consensus on what the problem is and how it should be addressed (problem definition; Shiffman 2017). Although the dental community agrees that oral health is integral to general health and essential for general well-being and quality of life (Glick et al. 2017), it has not been able to articulate key definitions of the causes of and the solutions to those problems. There is a need to recognize the importance of the social and commercial determinants of health that act globally to generate oral health inequalities. There is a disjoint in terms of solutions too. While a few push for upstream action (taxation and fiscal policies) to address oral health inequalities (Watt et al. 2019), much research work and practice remain downstream, particularly focused on dental health education and complex biomedical interventions of questionable cost-effectiveness (Stein et al. 2018). What is more, most dental care services around the world are delivered privately; thus, they are business oriented, with a wide disparity in available oral health resources between high- and low-/middle-income countries.

The second challenge is to portray the issue in ways that inspire external audiences to act (positioning). This is a problem of external framing (Shiffman 2017). We must convey oral health issues in ways that resonate with external players whose resources are needed to address health problems. Traditional population dental metrics are not obvious beyond dentistry. Indices such as the DMFT are known for their limitations but continue to be used. By using comparable metrics such as years lived with disability, the GBD study provided evidence that the burden of dental diseases is comparable to the burden of all maternal conditions combined, hypertensive heart disease, anxiety disorders, and schizophrenia and greater than 25 of the 28 categories of cancer, cardiovascular and cerebrovascular diseases, and mental health other than depression (Kassebaum et al. 2017). However, not everybody in the dental field is familiar with these metrics or knows how to use them to advocate for the relevance of oral health.

The third challenge is to forge alliances with these external actors (coalition building), especially those outside the health sector (Shiffman 2017). Most dental organizations—such as the International Association for Dental Research, World Dental Federation, and World Health Organization (WHO) global oral health unit—work in isolation with little alignment in terms of policy direction. Important steps toward coalition building include the inclusion of oral health into the Political Declarations of the High-Level Meetings on the prevention and control of noncommunicable diseases and universal health coverage (UHC; i.e., access to essential quality health services without financial hardship) (UN General Assembly 2011, 2019) as well as the statement of the World Federation of Public Health Associations (2016) supporting the integration of oral health into primary health care and public health systems.

The fourth challenge is to establish institutions to facilitate collective action (governance). Depending on the kind of leadership agreed among members, global health networks could be shared (equal rights to all members), led (a single coordinator), and network administrative organizations (members represented by a board that makes the decisions; Shiffman 2017). There are not generally accepted individual leaders in dentistry, and this fragmentation is a barrier to collective action. It is worth reiterating that such a global network should consist of dental and nondental members with good representation from all parts of society, including those influencing health priorities and resource allocation. A major challenge for wider engagement is inclusion. The main actors do not want to get out of the silos in which they are comfortable.

How well our profession addresses these 4 challenges will shape our performance during the SDG era and beyond. The SDG agenda offers an opportunity to start aligning our efforts to global health. Although oral health is not specifically mentioned in the 13 targets of SDG 3 (good health and well-being), these targets include cardiovascular, cancer, diabetes, and respiratory outcomes (3.4.1); essential health service coverage (3.8.1); and tobacco use (3.a.1; UN General Assembly 2015). Dental professionals may play a significant role to achieve SDG 3 because oral health promotion addresses risk factors common to oral conditions and these noncommunicable diseases. Other critical areas of work relate to SDG 6 (clean water and sanitation), as water fluoridation is the most cost-effective population approach to address the burden of dental caries and its unequal distribution, which addresses SDG 10 (reduced inequalities; UN General Assembly 2015). The advancement of oral health cannot be achieved by individual players or small dental teams working on their own. The complex causes of inequalities in health mean that intersectoral action is required in public health to tackle macroeconomic factors and the physical and social environment, as well as the adverse health behaviors and poor access to health care (Knai et al. 2018; Marmot and Bell 2019).

## Research Priorities

The development of a global health network for oral health requires addressing some fundamental gaps in knowledge, particularly in 3 areas of oral health action (Table). The first gap in knowledge is in global oral epidemiology and health information systems. Continuation and improvement of community oral health surveys and surveillance are crucial to ensure that timely, relevant, and current data are available for compilation and analyses. Concurrently, it is crucial to improve reporting of data and develop a database repository to facilitate the identification of oral health survey reports. Most scientific journals do not publish survey reports; thus, it is difficult to find this relevant information. Furthermore, we need to develop an analytic framework that leverages the interconnectedness of oral conditions to improve the degree to which incomplete data can generate actionable estimates of oral health burden worldwide. The WHO Oral Health Country/Area Profile Project must be improved and follow a systematic approach for selection, appraisal, and reporting of data sources. The GBD study offers a systematic and comprehensive approach to data management, leading the way on oral health burden estimation, but it is not without criticism (Shiffman and Shawar 2020). The GBD study needs primary data of good quality to calculate health estimates. Addressing this gap should start by developing an appropriate global health information system to serve evidence-based planning and monitoring. Also, we need to transparently delineate ideal and “alternate” reporting metrics, with reporting methods that everybody—not only dentists and dental researchers—can understand, readily put into practice, and describe to others. The result would be a data repository to provide researchers and policy makers the evidence needed for planning, implementation, and evaluation of oral health policies and dental health care.

**Table.** Research Priorities to Strengthen Global Health Action for Oral Health.

Gaps in Knowledge	Research Priorities
1. Epidemiology and health information systems for surveillance of oral conditions	1.1. Consolidate methods for data collection and reporting, preferably at a person level (such as prevalence, incidence, years lived with disability, and disability-adjusted life years) rather than a tooth or surface level
	1.2. Conduct population-based surveys reporting oral epidemiology data across all age groups (not just children), especially in low- and middle-income countries where such information is lacking
	1.3. Monitor the extent of absolute and relative social inequalities in oral health, especially in low- and middle-income countries
	1.4. Develop robust methods to convert traditional oral epidemiology indices into person-level estimates of untreated disease for burden estimation
2. Collection, harmonization, and rigorous assessment of evidence for equity in prevention and treatment of oral conditions	1.5. Evaluate the relative importance of environmental, socioeconomic, commercial, and behavioral risk factors on the burden of oral conditions
	1.6. Identify health policies and interventions that can reduce inequalities in health and oral health simultaneously
	1.7. Evaluate the impact of existing (or about to be implemented) health policies on oral health by using quasi-experimental designs
3. Strategies to deliver essential quality oral health care without financial hardship	1.8. Revisit the dental curricula and develop novel educational methods to promote the incorporation of a social and commercial determinants of oral health inequalities perspective
	1.9. Evaluate the effectiveness, cost-effectiveness, and successful implementation of different oral care packages, based on health promotion, disease prevention, and minimal intervention dentistry.
	1.10. Operationalize appropriately comprehensive health economic approaches to prioritize choices about human resource and treatment technology inputs.
	1.11. Develop planning models of human resources and health care services aligned with current and future oral health care needs.

The second gap in knowledge relates to the collection, harmonization, and rigorous assessment of evidence for equity in prevention and treatment. A quantitative understanding of the drivers of disease at the population level is needed, including the role of social and commercial determinants of health; environmental, metabolic, and behavioral risk factors; and the effectiveness of interventions for prevention and treatment. The GBD study has quantified the connection between many risk factors and diseases, but research based on other study designs is also required. Randomized controlled trials (RCTs) have been criticized for evaluation of health promotion and policy (Frieden 2017). While RCTs or quasi-experimental designs are recommended to ascertain determinants of health and test the effectiveness of policy- or population-level interventions (Shadish and Cook 2009; West et al. 2008), attention must also be paid to the difficulties of implementing an effective intervention in the real world (Brown et al. 2017). Optimal adherence to an intervention is a key determinant of its success. Research designs that permit assessment of health promotion interventions in their natural settings for longer periods provide evidence of effectiveness as important as evidence from RCTs. Implementation research has the potential of identifying barriers and solutions to the successful adoption of an intervention. Finally, many countries have now implemented sugar and tobacco taxes, smoking bans, and food labeling. Formal impact assessments on oral health must be completed through appropriate research methods.

The third gap in knowledge relates to optimal strategies for delivering essential quality oral health care to all who need it (3.5 billion people) without imposing personal and public financial hardship. This includes but is not limited to the following: 1) revisiting dental curricula and educational methods, 2) building interprofessional and intersectoral teams to develop competency frameworks that help policy makers tackle the social and commercial determinants of health at all levels, 3) identifying strategies to incorporate social policies into health systems, and 4) evaluating the impacts of these changes on population oral health. Beyond addressing the broader determinants of oral health through upstream actions, we must ameliorate the suffering caused by the global burden of untreated oral conditions. To achieve this goal, dental care should be integrated into primary health care with a focus on minimal intervention dentistry, which is a groundbreaking biological medical approach focusing on the prevention and interception of the disease at an early stage and on health promotion. Universal coverage for oral health care based on conventional dental care (surgical) may be too expensive to tackle the current 3.5 billion cases of untreated dental conditions. The 2 principles of UHC (essential quality health services and financial protection; WHO and World Bank 2017) must be resolved if oral health care is to be adopted as part of UHC initiatives. The WHO Basic Package of Oral Care—which includes oral urgent treatment, affordable fluoride toothpaste, and atraumatic restorative treatment—is a good starting point, especially at the level of primary health care (Frencken et al. 2002). However, evidence of its successful implementation, sustainability, and scaling up is lacking (Chher et al. 2009). The role of delegating tasks (optimal skill mix) for the delivery of such packages deserves attention. This package could be expanded to include a range of cost-effective minimally invasive interventions. This approach is in line with the WHO’s best-buys approach wherein population interventions are ranked and recommended per their level of evidence and cost-effectiveness (WHO 2017). Solid evidence on economic evaluation and implementation success of oral health care packages addresses the first principle of UHC (providing essential quality oral health care; WHO and World Bank 2017; Righolt et al. 2020). The second UHC principle (financial protection) can be measured by using standard indicators such as catastrophic health expenditure and impoverishment, as demonstrated elsewhere (Masood et al. 2015; Bernabé et al. 2017). In addition, the modeling of oral health investment cases, as recently demonstrated in Burkina Faso (Jevdjevic and Listl 2020), can help identify programs that provide good value for money, with the required type, mix, and quantity of dental workforce. Successful and unsuccessful findings should be shared openly so that we can build up on experience. Countries such as Thailand and Brazil have been praised for achieving UHC, including essential dental care within primary care networks (Yiengprugsawan et al. 2010; Pucca et al. 2015), and they provide a good model for organization and delivery.

## Concluding Remarks

The GBD study showed that oral health has not significantly improved over the last 3 decades throughout the world and remains a major global public health challenge. Clearly, greater efforts and potentially different approaches are needed to promote global oral health. Conventional dental care is characterized by high cost, which may explain why there are 3.5 billion cases of oral conditions worldwide needing attention, why oral health has been neglected, and why dental care has not been included in UHC. We argue that a global health network for oral health must be harnessed to drive health system reform. The complexity of developing a new cost-effective oral health system, reducing oral health inequities, and integrating oral health into the health agenda requires a broader participation. The WHO may lead the way forward, build coalitions with relevant external actors, and create an inclusive and unifying global oral health network consisting of dental and nondental members with good representation from all parts of society to reach consensus on how to address the problem. The inclusion of multi-institutional dental health organizations, such as the International Association for Dental Research, World Dental Federation, and influential external actors in dentistry, is crucial for this effort to be successful. Such a network can approve and update (if needed) the research strategies proposed here. The GBD should independently monitor progress. The major indicator of success would be a reduction of the number of cases of untreated dental conditions. Oral health policies and dental care should be solidly based on scientific evidence. Health services must continue carrying out and improving the quality of oral health surveys and surveillance to ensure that timely, relevant, and current data are available for compilation and analyses. Oral health researchers must carry out rigorous assessment of evidence for equity in prevention and management of oral conditions. Finally, oral health care needs to move toward more cost-effective approaches to deliver care to all.

## Author Contributions

F.N. Hugo, N.J. Kassebaum, W. Marcenes, E. Bernabé, contributed to conception and design, drafted and critically revised the manuscript. All authors gave final approval and agree to be accountable for all aspects of the work.
